# Multi-tissue transcriptome profiling linked the association between tissue-specific circRNAs and the heterosis for feed intake and efficiency in chicken

**DOI:** 10.1016/j.psj.2024.103783

**Published:** 2024-04-20

**Authors:** Jingwei Yuan, Qin Li, Yanyan Sun, Yuanmei Wang, Yunlei Li, Zhangjing You, Aixin Ni, Yunhe Zong, Hui Ma, Jilan Chen

**Affiliations:** Key Laboratory of Animal (Poultry) Genetics Breeding and Reproduction, Ministry of Agriculture and Rural Affairs, Institute of Animal Sciences, Chinese Academy of Agricultural Sciences, Beijing 100193, China

**Keywords:** Chicken, Feed efficiency, Heterosis, CircRNA

## Abstract

Heterosis has been widely utilized in chickens. The nonadditive inheritance of genes contributes to this biological phenomenon. However, the role of circRNAs played in the heterosis is poorly determined. In this study, we observed divergent heterosis for residual feed intake (**RFI**) between 2 crossbreds derived from a reciprocal cross between White Leghorns and Beijing You chickens. Then, circRNA landscape for 120 samples covering the hypothalamus, liver, duodenum mucosa and ovary were profiled to elucidate the regulatory mechanisms of heterosis. We detected that a small proportion of circRNAs (7.83–20.35%) were additively and non-additively expressed, in which non-additivity was a major inheritance of circRNAs in the crossbreds. Tissue-specific expression of circRNAs was prevalent across 4 tissues. Weighted gene co-expression network analysis revealed circRNA-mRNA co-expression modules associated with feed intake and RFI in the hypothalamus and liver, and the co-expressed genes were enriched in oxidative phosphorylation pathway. We further identified 8 nonadditive circRNAs highly correlated with 16 nonadditive genes regulating negative heterosis for RFI in the 2 tissues. Circ-ITSN2 was validated in the liver tissue for its significantly positive correlation with *PGPEP1L*. Moreover, the bioinformatic analysis indicated that candidate circRNAs might be functioned by binding the microRNAs and interacting with the RNA binding proteins. The integration of multi-tissue transcriptome firstly linked the association between tissue-specific circRNAs and the heterosis for feed intake and efficiency in chicken, which provide novel insights into the molecular mechanism underlying heterosis for feed efficiency. The validated circRNAs can act as potential biomarkers for predicting RFI and its heterosis.

## INTRODUCTION

Feed cost accounted for more than 60% of total cost in the poultry production. Thus, feed efficiency is always attracting breeders and researchers to devote themselves to it. A deeper understanding of feed efficiency is critical at many levels. It impacts profitability of farmers, represents competitive position of chicken protein against other protein sources, and means less demand on global feed resources, as well as other environmental and societal implications (Patience et al., 2015). Generally, pedigree and genomic selection and cross breeding were the main breeding methods in chicken. Feed efficiency was largely improved though improving meat or egg production traits and using the heterosis of traits in the past decade (Siegel, 2023). The basis of crossbreeding is utilization of the heterosis from optimal parents’ pair, generating crossbreds with superior growth rate, production, or any other trait over its parents. However, screening parental lines using phenotypic data is always a laborious and time-consuming work. Based on findings from high-throughput omics, such as genomics, transcriptomes, epigenomics, and proteomics analysis, the reliable molecular markers have gradually been applied to the process of crossbreeding, accelerating the formation of better lines in plant (Hasan et al., 2021). Theoretically, inheritance model including dominance, overdominance, heterozygosity, epistasis, nonallelic interaction model explained the phenomenon of heterosis (Lippman and Zamir, 2007), which has been exploited till today. Although we revealed the molecular mechanism of heterosis for feed intake and efficiency using genome-wide expression data (Yuan et al., 2023), the underlying molecular mechanisms are remained elusive in chickens regarding the non-coding RNAs.

Circular RNAs (**circRNAs**) are recognized as a class of non-coding RNAs (**ncRNAs**) formed by the nonsequential back-splicing of pre-messenger RNAs. They have been identified widespread in eukaryotic tissues and cells from mammal (Yu and Kuo, 2019), model organism (Memczak et al., 2013), plant (Zhang and Da, 2022), and livestock (Robic et al., 2021). CircRNAs can arise from exons, introns, untranslated regions, ncRNA loci as well as intergenic and antisense transcripts. Unlike to the linear RNA counterpart, the circRNA forms a covalently closed continuous loop displaying stability and are not easily degraded by RNase R (Vromman et al., 2021), leading circRNA's application as biomarkers in the clinical and genetic use (Zhang et al., 2018), and ncRNA-related therapeutics targeting cancers, diabetes and hepatitis C have entered clinical trials (Rupaimoole and Slack, 2017). Increasing number of studies have demonstrated that circRNAs are acting as mediators, including miRNA sponges, anchors for circRNA binding proteins (**cRBPs**), transcriptional regulators, molecular scaffolds, and sources for translation of small proteins/peptides modulating the gene expressions, whose alterations were the basis of various phenotypes and diseases (Misir et al., 2022). Genome-wide gene and ncRNAs expression have been reported to be associated with heterosis in plant (Zhang et al., 2023a; 2023b) and animals (Wang et al., 2023). For example, nonadditively expressed small RNAs regulated heterosis for maize plant height through activating and suppressing genes involved in vegetative growth-related pathways and stress response pathways (Zhang et al., 2023a). Compared to the linear transcripts, the expression of circRNAs was more likely to be nonadditive in the maize hybrid (Luo et al., 2020). Shu et al (2021) reported that circRNAs might be involved in the heterosis for pepper growth by analyzing transcriptome of pepper leaves. However, whether circRNAs affect expression of related genes and traits heterosis is rarely reported in chickens.

Recently, circRNAs have emerged as integral components of folliculogenesis in the ovary (Shen et al., 2020), muscle development in the breast (Ouyang et al., 2018), parasites resistance in the cecum (Zheng et al., 2019) as well as spleen (Wang et al., 2020) and liver (Zhang et al., 2017) tumorigenesis, highlighting their versatile role played in chickens. Furthermore, the liver circRNAs targeted *RTP4* regulating residual feed intake (RFI) in Hu lamb (Zhang et al., 2022). Zhao et al (2023) found that the hypothalamic circRNAs regulated phenotypic variation of RFI via sponging miRNAs and regulatory pathways related to nutrient metabolism in cattle. Despite accumulating evidence for an important role of circRNAs in chicken and feed efficiency in animals were reported, our current understanding of their expression in modulating feed efficiency and the heterosis is lacking. Therefore, a robust large-scale dataset of circRNA expression across multiple tissues is needed.

Previously, we constructed a reciprocal cross population using 2 distinct breeds, and observed significantly divergent heterosis between 2 crosses (Zhao et al., 2022). Through analyzing the genome-wide gene expression of multiple tissues, we demonstrated that the oxidative phosphorylation (**OXPHOS**) pathway played a fundamental role in modulating the feed efficiency and the heterosis (Yuan et al., 2023). Nevertheless, the role of ncRNA, especially circRNA in the population and feed efficiency is still puzzling us. Herein, by using the strand-specific RNA-seq data, we profiled the circRNA expression across the hypothalamus, liver, duodenum mucosa and ovary tissue, and linked the profile to the feed efficiency and the heterosis, and established circRNAs and circRNAs-related gene regulatory networks affected the feed efficiency and the heterosis.

## MATERIALS AND METHODS

### Ethics Statement

All birds sampled in the study were approved by the guidance of ethical regulations from the Laboratory Animal Welfare and Animal Experiment Ethics Committee of the Chinese Academy of Agricultural Sciences (**IAS-CAAS**), Approval Code: IAS2022-35.

### Reciprocal Cross Population

The experimental population was derived from reciprocal crossing between White Leghorn (**WW**) and Beijing You chicken (Chinese indigenous breed, YY). The full mating procedure was previously reported (Yuan et al., 2023). Briefly, 30 YY roosters with similarly healthy status were randomly mated with 150 YY hens and 150 WW hens to generate purebred YY and crossbred YW, respectively. Thirty WW roosters with healthy status were randomly mated with 150 different WW hens and 150 different YY hens to generate purebred WW and crossbred WY, respectively. A 2-generation pedigree contained 60 parental birds and 417 maternal birds and 2,550 hens from the F_1_ generation was generated in the same hatch. At 18 wk of age, a total of 1,260 birds were transferred to individual cages in the same house providing ad libitum access to water and a commercial corn–soybean diet that met National Research Council requirements.

### Phenotypes and Heterosis

Feed intake was individually collected during a 4-wk (43–46 wk of age) trial period. The detailed operations were described previously (Yuan et al., 2023). We recorded the feed consumption, egg number and weight, body weight, and then calculated daily feed intake (**DFC**), daily egg mass (**DEM**), metabolic BW (BW raised to the power of 0.75) and daily body weight gain (**BWG**). Feed conversion ratio (**FCR**) was calculated as a ratio of DFC and DEM. A total of 904 hens including 198 WW, 245 YY, 238 YW, and 223 WY that laid eggs in the feeding trial were used to calculate residual feed intake (**RFI**) as the residuals from a regression model of DFC on metabolic BW (MBW), BWG and DEM (Yuan et al., 2015) using lm() function in R software. Then, mid-parent heterosis was calculated according to the equation:

(1) $$H=\;\frac{\bar{F}-\left(\bar{P_{w}}+\;\bar{P_{y}}\right)/2}{\left(\bar{P_{w}}+\bar{P_{y}}\right)/2}$$

where $\bar{F}$, $\bar{P_{w}}$and $\bar{P_{y}}$ represent the average value of traits for the reciprocal crosses, WW and YY, respectively. Then, a transformed Student's t test value was estimated to test the significance of heterosis based on the formula below (Mai et al., 2021):

(2) $$t=\;\frac{H}{2\sqrt{\frac{\sum {\left(F_{i}-\bar{F}\right)}^{2}}{n-1}}/\left[\left(\bar{P_{w}}+\bar{P_{y}}\right)\times \sqrt{n}\right]}$$

where $F_{i}$ is the phenotype of ith bird from reciprocal crosses; n is the number of WY birds or YW birds. The significance (*P* value) of heterosis was calculated using the pt() function in the R software according to the t value and the degrees of freedom, and the value less than 0.05 was considered significant.

### Sample Collection and Sequencing

Eight hens with RFI value around the population mean of each group from different family were euthanized by cervical dislocation for sampling tissues. Total RNA of hypothalamus, liver, and duodenum mucous were isolated using the TRIzol® Reagent (Invitrogen, Carlsbad, CA) according to the manufacturer's instructions. A total of 96 qualified RNA samples including 31 hypothalamus, 33 liver and 32 duodenum mucosae were used for Ribo-minus strand-specific RNA sequencing to generate 150 bp paired-end sequences on the Illumina nova 6000 platform (Illumina Inc., San Diego, CA). In birds, the liver is the main site for carbohydrate metabolism, fat production, and protein synthesis, and plays an important role in controlling the nutritional intake and food intake, converting nutrients into muscle and adipose tissue (Zaefarian et al., 2019). The intestine is the main site for nutrient absorption and digestion, while the duodenum accounts for over 50% of the digestive body (Denbow, 2015). Differential gene expression analysis also identified multiple candidate genes associated with high and low RFI in the duodenum, confirming the significant role of the duodenum in regulating feed efficiency compared to other intestinal segments (Yi et al., 2015). The hypothalamus is the core for regulating appetite, feed intake, and maintaining energy balance. In chicken population selected for high and low RFI, researchers found that the differential expression of neuropeptides such as hypothalamic SOCS3, growth hormone releasing peptide receptor, appetite stimulating neuropeptide Y, and Agouti-related proteins was significantly correlated with divergent RFI (Sintubin et al., 2014). Additionally, 6 ovarian samples each from 4 genetic groups were included in the RNA-sequencing for comparison purpose among tissues. The clean reads that filtered by following parameters: 1) containing adaptors; 2) with more than 10% unknown nucleotides; 3) with more than 50% low-quality bases (Qphred ≤20), were used for the circRNA identification. The RNA-seq data sets have been deposited in the National Genomics Data Center database(https://ngdc.cncb.ac.cn/, NGDC) with the BioProject number PRJCA012606 and PRJCA021760.

### CircRNA Identification and Characteristics Analysis

CircRNAs were predicted and screened followed the pipeline as Fig. S1. In the find_circ, clean data were first mapped to the chicken reference genome (**GRCg6**) using bowtie2 (v2.4.5) (Langmead and Salzberg, 2012). Then, the samtools (v1.12) was applied to extract the unmapped reads, which were split into anchor reads using unmapped2anchors.py script. Next the paired ordering anchors were aligned individually to the reference genome. The resulting alignments were handled by a customized script that jointly evaluates consecutive anchor alignments belonging to the same original read, performs extensions of the anchor alignments, and collects statistics on splice sites. Based on the statistics, we kept circular RNAs, satisfying at least 2 reads with unambiguous detection of the breakpoint and unique anchor alignments on both sides of the junction and splice sites less than 100 kb (Memczak et al., 2013). In the CIRI2, the clean data were mapped to the reference genome using bwa (0.7.17) (Li and Durbin, 2009). Then the sequence alignment map (**SAM**) files were scanned twice to collect sufficient information for identifying and characterizing circRNAs (Gao et al., 2015). The circRNAs identified by both algorithms were fed to the CIRIquant (v1.1) (Zhang et al., 2020) for the quantification. The expression was calculated by counts per million mapped reads (**CPM**). Expressed circRNAs were defined as CPM > 0.01 in at least one sample (Gruhl et al., 2021), and tissue-expressed circRNAs were those expressed in more than 30% of samples in one tissue, and circRNAs expressed in at least half of all samples were considered highly expressed among tissues. The CircAtlas database (Wu et al., 2020) was retrieved to tell the known circRNA and novel circRNA apart. Moreover, we compared the conservation scores of circRNAs, upstream 2 kb sequences and downstream 2 kb sequences using phyloP. The conservation scores were calculated as the average scores of all sites (Rybak-Wolf et al., 2015). For single parent expressed (**SPE**) circRNAs (expressed only in one of the parents), transcripts having CPM ≥ 1 in WW and CPM < 0.1 in YY were denoted SPE_W, and those with CPM < 0.1 in WW and CPM ≥ 1 in YY were denoted SPE_Y (Luo et al., 2020).

### Differential Expression and Pattern Analysis

The back spliced junction (**BSJ**) count calculated by CIRIquant was “regularized log” transformed for principal component analysis (**PCA**), which was visualized using the DESeq2 (v.1.16.1) (Love et al., 2014). The BSJ counts were fed to the DESeq2 (v.1.16.1) to carried out the differential expression analysis among 4 groups using the following contrast: WW vs. YY, WY vs. WW, WY vs. YY, YW vs. WW and YW vs. YY. CircRNAs with P value < 0.05 and | Log2 (fold change) | > 1.0 were considered differentially expressed in the corresponding contrast. The expression patterns of circRNAs were divide into additivity, dominance and overdominance based on the *P* value and Log2 (fold change) (Table S1) (Swanson-Wagner et al., 2006). The additivity was considered when the expression was significantly (*P* value < 0.05) different between WW and YY, and the expression of reciprocal crosses (WY or YW) was equal to (*P* value ≥ 0.05) the average of WW and YY. The positive and negative Log2 (fold change) between WW and YY were defined as additivity IV and X, respectively. Expression in WY or YW that was not significantly different from that in WW but significantly (*P* value < 0.05) lower and higher than that in YY was regarded as dominance V and XI, respectively. Expression in WY or YW that was not different from that in YY but significantly (*P* value < 0.05) higher and lower than that in WW was regarded as dominance III and IX, respectively. Expression in WY or YW that was significantly (*P* value < 0.05) higher (or lower) than that in both WW and YY was defined as overdominance including above high-parent pattern (II and XII), above parent pattern (I) or underdominance including below low-parent pattern (VI and VIII), below parent pattern (VII).

### Weighted Gene Co-expression Network Analysis

The co-expression modules were separately identified using normalized expression of circRNAs and genes for the hypothalamus, liver, duodenum mucosa and ovary performed in WGCNA (Langfelder and Horvath, 2008). Firstly, circRNAs with median absolute deviation (**MAD**) lager than 0 and genes with MAD ranking top 25% were used to construct expression matrix. The MAD was calculated by mad () function using R. Then, an appropriate “soft-thresholding” value was calculated using pickSoftThreshold() function for each tissue by plotting the correlation against a series of soft threshold values (from 1 to 30) under a signed pairwise correlation matrix generated by Pearson's method. If the powerEstimate was null, the value was further set to be 18, 16, and 14 if number of samples less than 20, 30, and 40, respectively. The correlation matrix was subsequently transformed into an adjacency matrix, in which node and edge corresponded to circRNA/gene and the degree of connection between circRNA and gene, respectively. Each adjacency matrix was normalized using a topological overlap function. Average linkage was applied to perform hierarchical clustering (Xu et al., 2021), and then the cluster tree was cut into modules that contained at least 50 circRNA/gene using the dynamic tree-cut algorithm. Modules with the correlation between their eigengenes greater or equal to 0.25 were merged. The eigengene is defined as the first principal component of their gene expression values. After obtaining modules, module eigengene was calculated with the “ModuleEigengenes” function. Correlation analysis between a module and the traits (MBW, BWG, DEM, DFC, and RFI) for each tissue was performed with function of “corPvalueStudent” based on the module eigengene, and *P* < 0.01 was set for the statistical significance.

### The Prediction of CircRNA-miRNA-mRNA Axes

We firstly identified circRNA-mRNA interactions which were considered if the circRNA expression was positively correlated with the corresponding mRNA expression (Spearman's *r* > 0.6 and *P* < 0.05). Then the nonadditively expressed circRNA-mRNA pairs harbored in the modules significantly associated with feed efficiency were used to predict microRNAs in the miRDB database (Wong and Wang, 2015). The target (predicted) sequence including 5′ untranslated region or coding sequence of unannotated genes were downloaded from Ensmbl database (https://apr2022.archive.ensembl.org/Gallus_gallus/Info/Index). MicroRNAs with target score larger than 60 and shared by circRNA-mRNA pairs were considered as candidate microRNAs. The candidate microRNAs were then evaluated for best binding sites and minimum free energy, and visualization in the RNAhybrid (Krüger and Rehmsmeier, 2006). To explore the protein coding potential of the circRNAs, we used the TransDecoder (https://github.com/TransDecoder/TransDecoder) and IRESfinder (Zhao et al., 2018) to predict the open reading frame (**ORF**) and internal ribosome entry site (**IRES**) of candidate circRNAs, respectively.

### Function Annotation and Network Visualization

Gene ontology (**GO**) enrichment analysis and Kyoto Encyclopedia of Genes and Genomes (**KEGG**) pathway analysis of candidate genes and host genes of circRNAs were performed using ClusterProfiler package implemented in R. False discovery rate (**FDR**) method was used to adjust the *P* values. GO terms and KEGG pathways with adjusted *P* value < 0.05 were significantly enriched. Illustration of circRNA-mRNA networks was plotted by the Cytoscape (https://cytoscape.org/).

### RNase R Digestion and qRT-PCR

Total RNA was extracted using the TRIzol® Reagent (Invitrogen) according to the manufacturer's guidelines. The extracted RNA was evaluated by 1% agarose gel electrophoresis. RNA purity, concentration, and integrity of all eligible RNA samples were determined using a NanoPhotometer® spectrophotometer (IMPLEN, CA). The cDNA was generated by reverse transcription using HiScript® III All-in-one RT SuperMix Perfect for qPCR (Vazyme, Nanjing, China). The stability of circRNA was determined using the Rnase R kit (Epicentre, Madison, WI) according to the manufacturer's guidelines. Briefly, 5 μg of total RNA was digested with 20 U RNase R for 35 min at 37°C and incubated at 80°C for 10 min. Then, the expression of circRNA and linear gene was quantified. For qRT-PCR reaction, the amplification system was established with reference to the instructions of Taq Pro Universal SYBR qPCR Master Mix (Vazyme, Nanjing, China). The qRT-PCR reaction was performed under the following conditions: pre-denaturation at 95°C for 2 min; 40 cycles of 95°C for 3 s; annealing temperature at 60°C for 32s; followed by a single melt cycle of 95°C for 15 s, 65°C for 1 min and 95°C for 15 s. All reactions were performed in triplicate for each sample. The relative expression levels of circRNA and gene were normalized to GAPDH using the 2-ΔΔCt method. In addition, divergent and convergent primers were designed for circRNA validation. The reaction mixture was pre-denatured for 3 min at 95°C, and then amplified for 35 cycles by denaturing for 30 s at 95°C, annealing for 30 s at 55°C, and extending for 30 s at 72°C, followed by final extension for another 5 min at 72°C. The PCR products were separated on 1.5% agarose gel electrophoresis. The primer sequences used in the present study were listed in Table S2.

## RESULTS

### Phenotypes and the Characterization of circRNAs

For the 2 purebreds and their reciprocal crosses, the average DFC ranged from 90.84 to 103.27 g/d, and the average DEM ranged from 36.85 to 52.85 g/d. The average RFI ranged from -3.10 to 3.70 g/d, and the average FCR ranged from 2.07 to 3.31. The crossbred YW showed significantly negative heterosis for both DFC and RFI, while crossbred WY showed significantly positive heterosis for RFI, FCR, and DEM. The details of phenotypic value were shown in Table S3.

**Figure 1:**
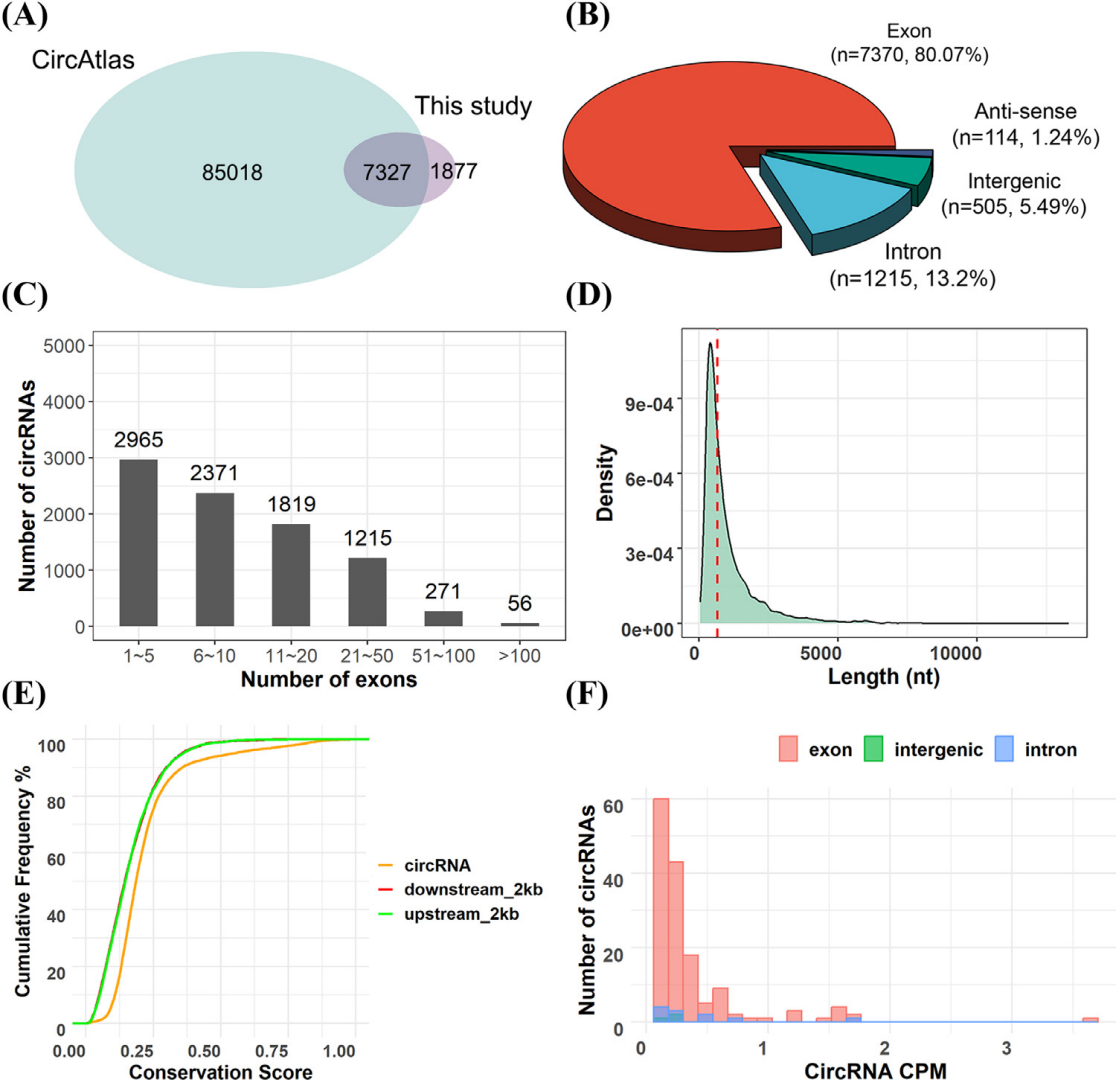
The characteristics of circRNAs in chicken. (A) the overlap between identified circRNAs in the current study and chicken circRNAs deposited in the circAltas database. (B) The type of circRNAs classified by the genomic regions. (C) The exon number distribution of identified circRNAs. (D) The length distribution of identified circRNAs. The red dash line denotes the median length 674 bp. (E) The conservation of CircRNAs and flanking 2kb sequences. (F) The expression distribution of different types of circRNAs. CPM denotes back splicing junction counts per million mapped reads.

Based on the average value of RFI, we sequenced the hypothalamus, liver, duodenum mucosa for 4 genetic groups with eight birds in each group. The RNA sequences of ovary samples from 24 birds (6 birds each in 4 genetic group) were also used in the present study to characterize the multi-tissue landscape of circRNA profiles in chicken. We analyzed 120 RNA-seq dataset and annotated 9,204 circRNAs that contained at least 2 unique back-spliced reads across the hypothalamus, liver, duodenum mucosa and ovary. Among these circRNAs, 7,327 circRNAs were reported in the circAtlas database, and 20.93% circRNAs were firstly reported in chickens (Figure 1A and Table S4). Most circRNAs located in exons (7,370), followed by intronic (1,215), intergenic (505), and anti-sense (114) circRNAs (Figure 1B). The characteristics analysis showed that circRNAs with exon number ≤10 accounted for over 50% of total circRNAs (Figure 1C), and the median length of the circRNAs were 674 bp (Figures 1D). The circRNA sequence and the upstream and downstream sequences of circRNAs within 2 kb were highly conserved with consistent phyloP score and cumulative frequency (Figure 1E), indicating that circRNA was stable and conserved. Furthermore, we measured the circRNA expression levels using CIRIquant by back splice junction counts per million mapped reads, which normalized the sequencing depths among samples. We found that different types of circRNAs were lowly expressed across 4 tissues (Figure 1F).

### CircRNA Expression Landscape in Parental Lines and Their Reciprocal Crosses

**Figure 2:**
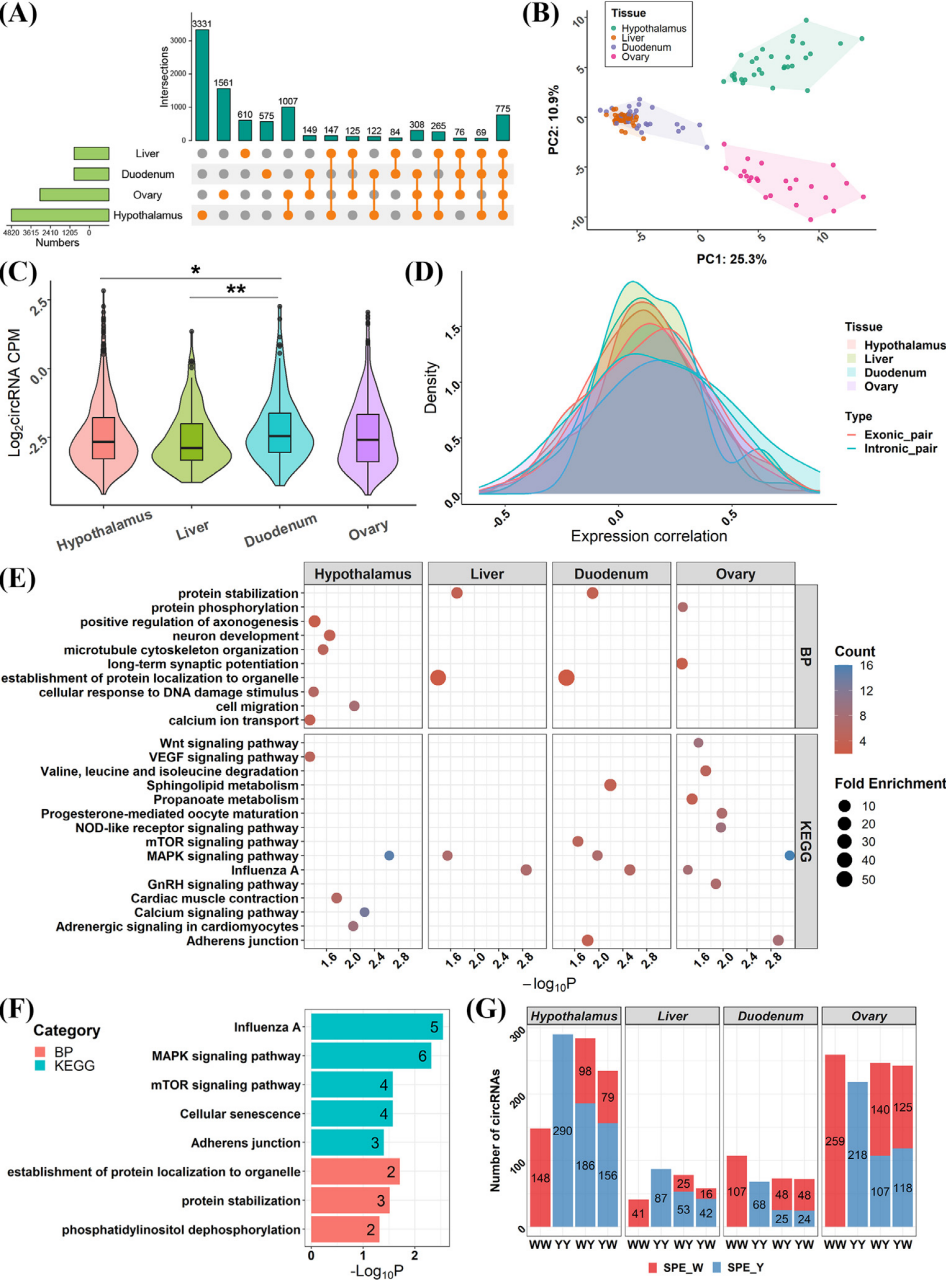
The comparison of identified circRNAs among hypothalamus, liver, duodenum mucosa and ovary. (A) The number of circRNAs identified in the 4 tissues. (B) The principal component analysis for the 4 tissues using circRNA expression data. (C) The comparison of circRNA expression among the 4 tissues. * *P* < 0.05, ** *P* < 0.01, Wilcox test. (D) Density diagram of correlation between circRNAs and host genes for the 4 tissues. The red and blue lines represent correlation of exonic circRNA-host gene pairs and intronic circRNA-host gene pairs, respectively. (E) Gene ontology biological processes and KEGG pathways that significantly enriched by host genes of circRNAs in the 4 tissues. (F) Gene ontology biological processes and KEGG pathways that significantly enriched by host genes of circRNAs that shared by the 4 tissues. (G) The number of single-parent expressed (**SPE**) circRNAs in the parents and crossbreds. SPE_W and SPE_Y denote circRNAs expressed only in White Leghorn and Beijing You chicken, respectively.

For the investigated tissues, the hypothalamus possessed most circRNAs (6,024) that expressed in at least one sample. The ovary, liver, and duodenum mucosa had less circRNAs containing 4,266, 2,151, and 2,158, respectively. CircRNAs showed a tissue-specific profile in the hypothalamus, in which the number of hypothalamus-specific circRNAs (3,331) accounted for over half of the total circRNAs (Figure 2A). Then, PCA showed tissue-specific clustering of the expressed circRNAs with MAD > 0 in the hypothalamus and ovary, while the liver and duodenum mucosa were intersected with each other and clustered separately from the hypothalamus and ovary (Figure 2B), suggesting that hypothalamus and ovary had a unique expression profile. Also, we compared the expression level of highly expressed circRNAs (expressed in at least half of all samples) among 4 tissues and found that the expression level of circRNAs in the duodenum mucosa was significantly higher than that of hypothalamus and liver (Figure 2C). We further extracted the host genes of highly expressed circRNAs for each tissue, obtaining 468, 138, 130, and 399 genes for hypothalamus, liver, duodenum mucosa and ovary, respectively (Table S5). Then, we calculated pair-wised Spearman correlation between exonic/intronic circRNAs and their host genes in each tissue. As shown in the Figure 2D, both exonic and intronic circRNAs would enhance the expression of their host genes. In comparison, circRNAs was more positively correlated with the expression of host genes, in which 86.48% of the exonic-circRNAs and host gene pairs and 95.65% of intronic-circRNA and host gene pairs showed a positive correlation with *p* < 0.05 in 4 tissues (Table S6). The function annotation showed that host genes were significantly enriched in biological processes including the calcium ion transport, neuron development, axonogenesis and so on in the hypothalamus. Protein stabilization and establishment of protein localization to organelle were enriched by host genes of liver and duodenum mucosa, and protein phosphorylation and long-term synaptic potentiation were enriched by host genes of ovary. Similarly, various KEGG pathways were enriched by host genes among 4 tissues (Figure 2E). Of note, host genes shared by 4 tissues were enriched in the protein stabilization process, MAPK signaling pathway, mTOR signaling pathway, adherens junction and so on (Figure 2F), suggesting the versatile role of these signaling pathways played in the tissues. For the single parent expressed (**SPE**) circRNAs in the hypothalamus and liver, more SPE circRNAs were detected in Beijing You chicken (**SPE_Y**), and also the reciprocal crosses expressed more SPE_Y circRNAs, while this trend is reversed for the duodenum mucosa and ovary, in which more SPE circRNAs were detected in White Leghorn chicken (**SPE_W**) and in the reciprocal crosses (Figure 2G). The results indicated that circRNAs were highly tissue-specific expressed compare to the genotype-specific expressed attributes.

### Tissue-Specific Expression Pattern of circRNAs in the Reciprocal Crosses

**Figure 3:**
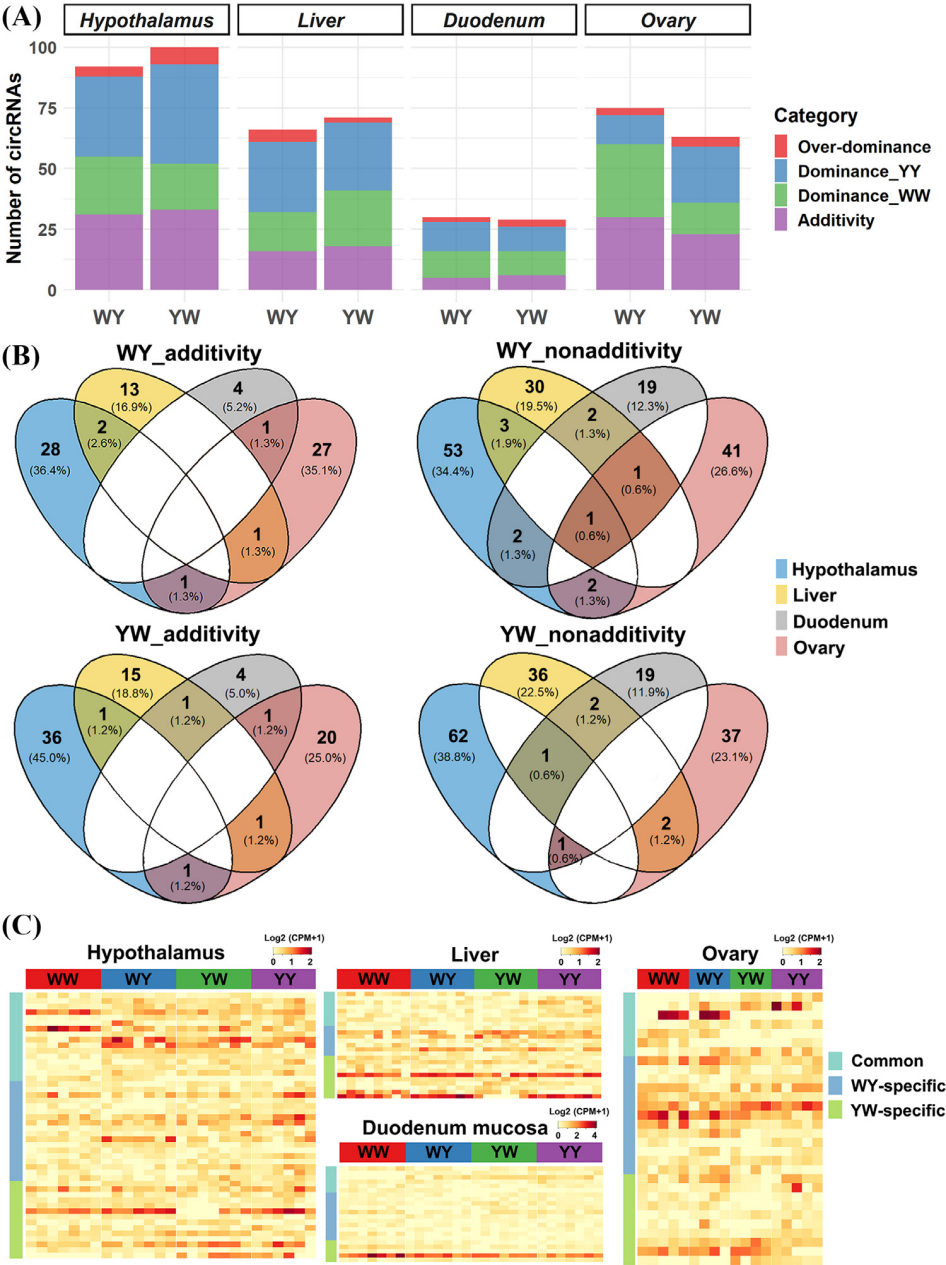
Inheritance pattern of circRNA expression in the hypothalamus, liver, duodenum mucosa and ovary in the crossbreds (WY and YW). (A) The proportion of additive, dominant, and over-dominant circRNAs in the 4 tissues. (B) The Venn plot for additive and non-additive circRNAs in the 4 tissues for the 2 crossbreds. (C) The expression heatmap for nonadditive circRNAs in the 4 tissues of parental line and reciprocal crosses.

Based on the theory that the nonadditive expression contributed to the heterosis, we conducted the circRNAs differential analysis and analyzed the expression patterns for highly expressed circRNAs in each tissue. As shown in Figure 3A, a small-scale circRNAs ranging from 7.83 to 20.35% were differentially expressed in the crossbreds (Table S1). Among these circRNAs, the nonadditive expressions including dominance and over-dominance, were major inheritance pattern across 4 tissues in the crossbreds. We identified 61, 50, 25, and 45 nonadditive circRNAs in the hypothalamus, liver, duodenum mucosa and ovary, respectively for WY. In YW, the number was 67, 53, 23, and 40 for the hypothalamus, liver, duodenum mucosa and ovary, respectively. In the dominance pattern, we compared the WW-biased (**WW_d**) and YY-biased dominance (**YY_d**) circRNAs in 4 tissues of crossbreds, and found that YY_d circRNAs were mainly expressed in the hypothalamus and liver. The number of WW_d and YY_d circRNAs was comparable between crossbreds in the duodenum mucosa, while more WW_d circRNAs expressed in WY than that expressed in YW for the ovary. By focusing on the additive and nonadditive circRNAs, we detected the common circRNAs among 4 tissues. We found that most circRNAs were separately owned by tissue-selves (Figure 3B). The tissue-specifically additive circRNAs accounted for 93.6% and 93.8% for WY and YW, respectively, and the tissue-specifically nonadditive circRNAs accounted for 92.8% and 96.3% for WY and YW, respectively, supported that circRNAs functioned in a tissue-specific way regulating the feed efficiency and its heterosis. Since the RFI and its heterosis were divergent between the crossbreds, we further classified the nonadditive circRNAs into breed-common and breed-specific type. We identified 16 common, 18 WY-specific and 14 YW-specific circRNAs in the hypothalamus, 8 common, 7 WY-specific and 10 YW-specific circRNAs in the liver, 6 common, 11 WY-specific and 5 YW-specific circRNAs in the duodenum mucosa, and 7 common, 13 WY-specific and 10 YW-specific circRNAs in the ovary, respectively (Figure 3C). For the common circRNAs, divergent expression pattern between crossbreds that might be associated with the divergent heterosis was screened in each tissue, such as gal-FOCAD_0001 in the hypothalamus, chr32:598357|600118 in the liver, gal-GCH1_0002 in the duodenum mucosa, and gal-CASP6_0002 and gal-ENSGALG00000000360_0003 in the ovary (Table S7). Moreover, 32, 17, 16 and 23 genotype-specific circRNAs were also detected in the hypothalamus, liver, duodenum mucosa and ovary, respectively (Table S7).

### The Co-expressed circRNA-mRNA Modules Related to the Feed Efficiency

**Figure 4:**
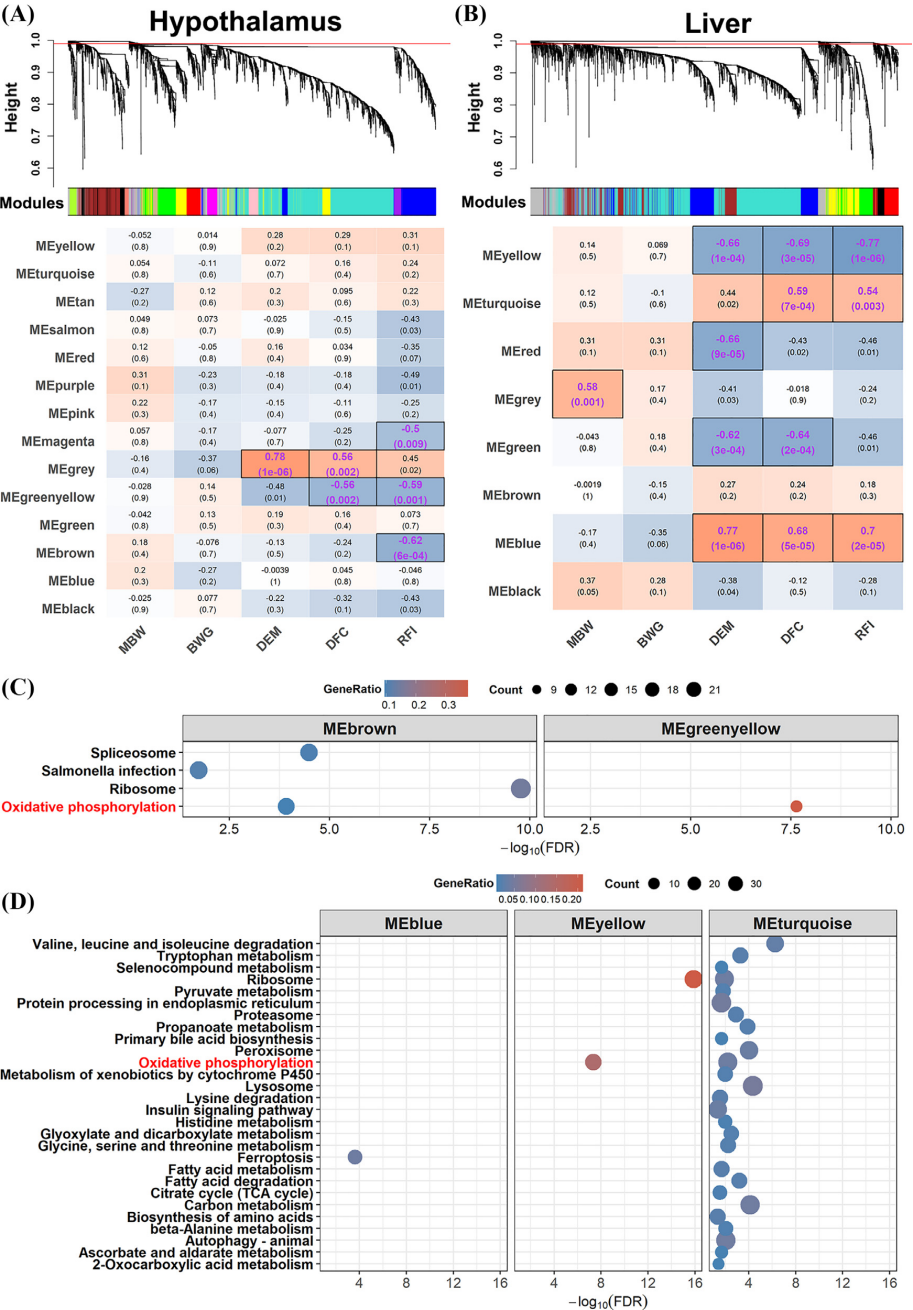
Weighted gene co-expression network analysis for the parental line and reciprocal crosses. The hierarchical cluster dendrogram and module-trait relationships were plotted for the hypothalamus (A) and liver (B), respectively. Heatmap colors indicate positive/negative Pearson correlation coefficients. Correlation coefficients and *P-*values are shown within the cells (purple font, *P* < 0.01). MBW metabolic body weight, BWG body weight gain, DEM daily egg mass, DFC daily feed intake and RFI residual feed intake. KEGG pathways significantly enriched by genes in the RFI-related modules for the hypothalamus (C) and liver (D), respectively.

In order to narrow the key circRNAs associated with the heterosis for RFI, we performed weighted gene co-expression network analysis (**WGCNA**) with the circRNA and mRNA transcriptomic atlas for each tissue. We firstly identified 14, 8, 14, and 6 co-expressed modules in the hypothalamus, liver, duodenum mucosa and ovary, respectively. By correlating these modules with RFI and relevant traits, we found brown module (MEbrown, *r* = -0.62, *p* < 0.01), MEmagenta (*r* = -0.50, *p* < 0.01) and MEgreenyellow (*r* = -0.59, *p* < 0.01) were correlated with RFI in the hypothalamus (Figure 4A). In the liver tissue, MEblue (*r* = 0.70, *p* < 0.01), MEturquoise (*r* = 0.54, *p* < 0.01) and MEyellow (*r* = -0.77, *p* < 0.01) were significantly correlated with RFI (Figure 4B). The MEgreenyellow (*r* = -0.56, *p* < 0.01) in the hypothalamus, and the three RFI-associated modules (MEblue: *r* = 0.68, *p* < 0.01; MEturquoise: *r* = 0.59, *p* < 0.01; MEyellow: *r* = -0.69, *p* < 0.01) in the liver were also significantly correlated with DFC. For the duodenum mucosa, MEpink was significantly associated with DFC, while no related module was found for the RFI (Figure S2a). As expected, there was no modules related to the feed efficiency in the ovary (Figure S2b). Meanwhile, some modules were correlated with RFI at the significant level of 0.05 in the hypothalamus (MEred), liver (MEred and MEgreen) and duodenum mucosa (MEbrown and MEpink) (Figure 4 and Figure S2a). We further performed enrichment analysis for genes in the modules that significantly related to RFI. A total of 14 biological processes were annotated for the MEturquoise in the liver (Table S8). Four and 28 KEGG pathways were identified for the hypothalamus and liver, respectively (Figures 4C and 4D). Notably, the genes harbored in the negatively RFI-related modules (MEbrown and MEgreenyellow in the hypothalamus, MEyellow in the liver) were enriched in the oxidative phosphorylation pathway, which were reported to be associated with heterosis of feed intake and efficiency in our previous study (Yuan et al., 2023). The genes belonging to the positively RFI-related modules including the MEblue and MEturquoise of the liver were involved in the ferroptosis, carbon, amino acid, and fatty acid metabolism. These results suggested the nonnegligible role of circRNAs played in the feed efficiency regulation through a tissue-specific way.

### CircRNA-mRNA Networks Associated With Heterosis for Feed Efficiency

**Figure 5:**
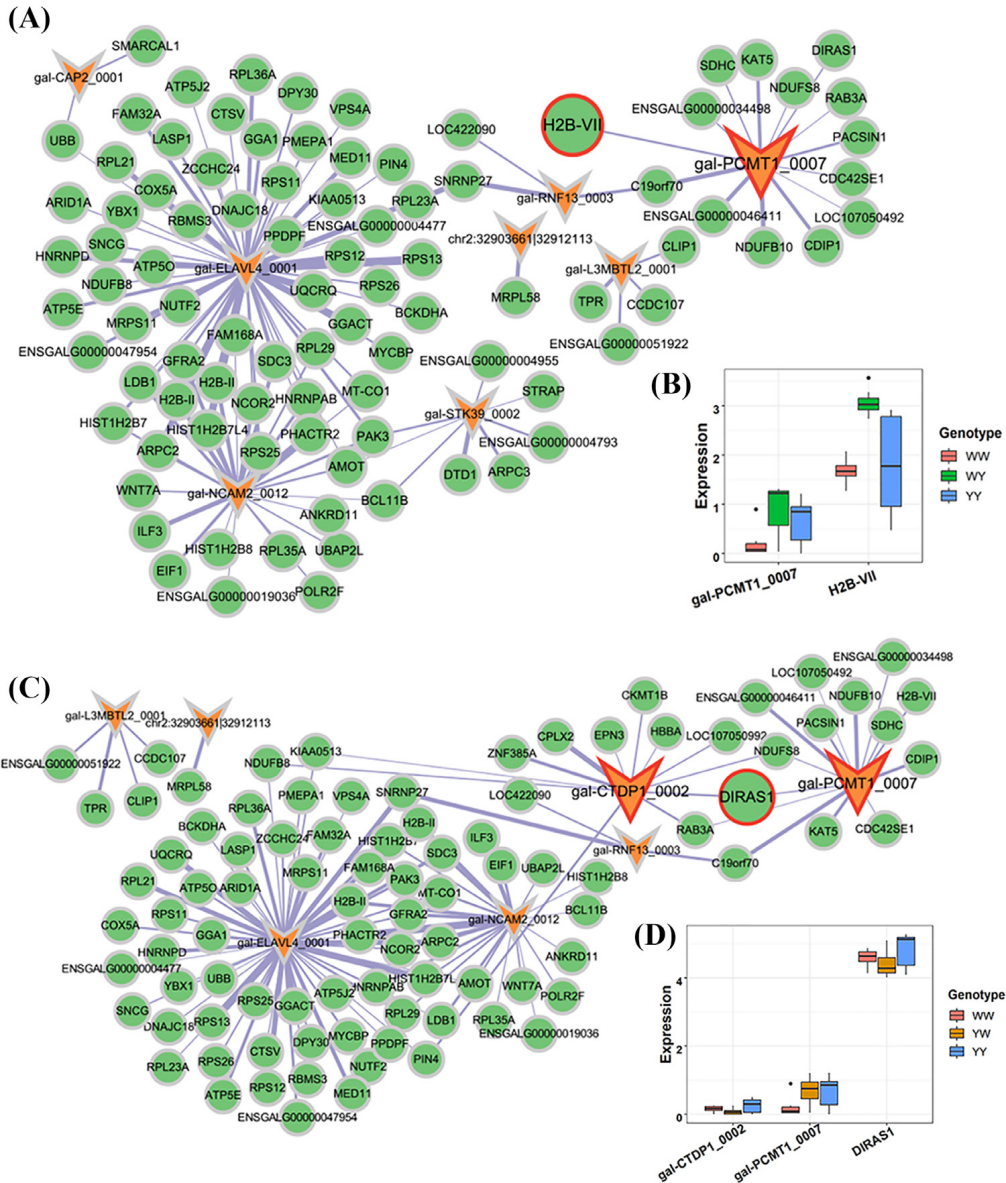
CircRNA-mRNA networks in the hypothalamus associated with heterosis for residual feed intake (**RFI**). (A) CircRNA-mRNA networks in the brown module that associated with RFI for WY. (B) The expression pattern of nonadditive circRNA and genes in WY. (C) CircRNA-mRNA networks in the brown module that associated with RFI for YW. (D) The expression pattern of nonadditive circRNA and genes in YW. In the networks, the circRNA and gene were plotted in “V” and “ellipse” shape, respectively. The shape with red border line denotes that the circRNA/gene is nonadditive expressed.

By focusing on the modules that significantly correlated with RFI, we constructed circRNA-mRNA networks that potentially regulated the heterosis for the hypothalamus and liver. In the hypothalamus, nonadditively expressed circRNAs were found in the MEbrown, in which 8 circRNAs were targeting to 93 genes in WY (Spearman's *r* > 0.6 and *P* < 0.05, Figure 5A). CircRNA gal-PCMT1-0007 was dominantly expressed interacting with 14 genes including one dominantly expressed gene *H2B-VII* (Figure 5B), which was affiliated to the histone cluster, playing a central role in transcription regulation, DNA repair, DNA replication and chromosomal stability. *H2B-VII* was expressed in same pattern as gal-PCMT1-0007 (Table S9a). For YW, we identified 93 genes were targeted by 7 circRNAs (Spearman's *r* > 0.6 and *P* < 0.05), in which gal-PCMT1-0007 and gal-CTDP1-0002 were dominantly expressed targeting 26 genes (Figure 5C). The 2 circRNAs trans-regulated the nonadditive gene *DIRAS1* (DIRAS family GTPase 1), which is involved in GTP binding and mitogen-activated protein kinase binding. *DIRAS1* was expressed in a below-parent pattern, which was same as the pattern of gal-CTDP1-0002 (Figure 5D and Table S9a). The gal-PCMT1-0007 showed same expression pattern between the 2 crossbreds, which was inconsistent with the divergent RFI between the 2 crossbreds. Therefore, the gal-CTDP1-0002 and DIRAS1 pair was selected as candidate pair in the subsequent analysis for YW. Furthermore, no any nonadditive circRNA-mRNA pair involved in the oxidative phosphorylation pathway was detected in the hypothalamus.

**Figure 6:**
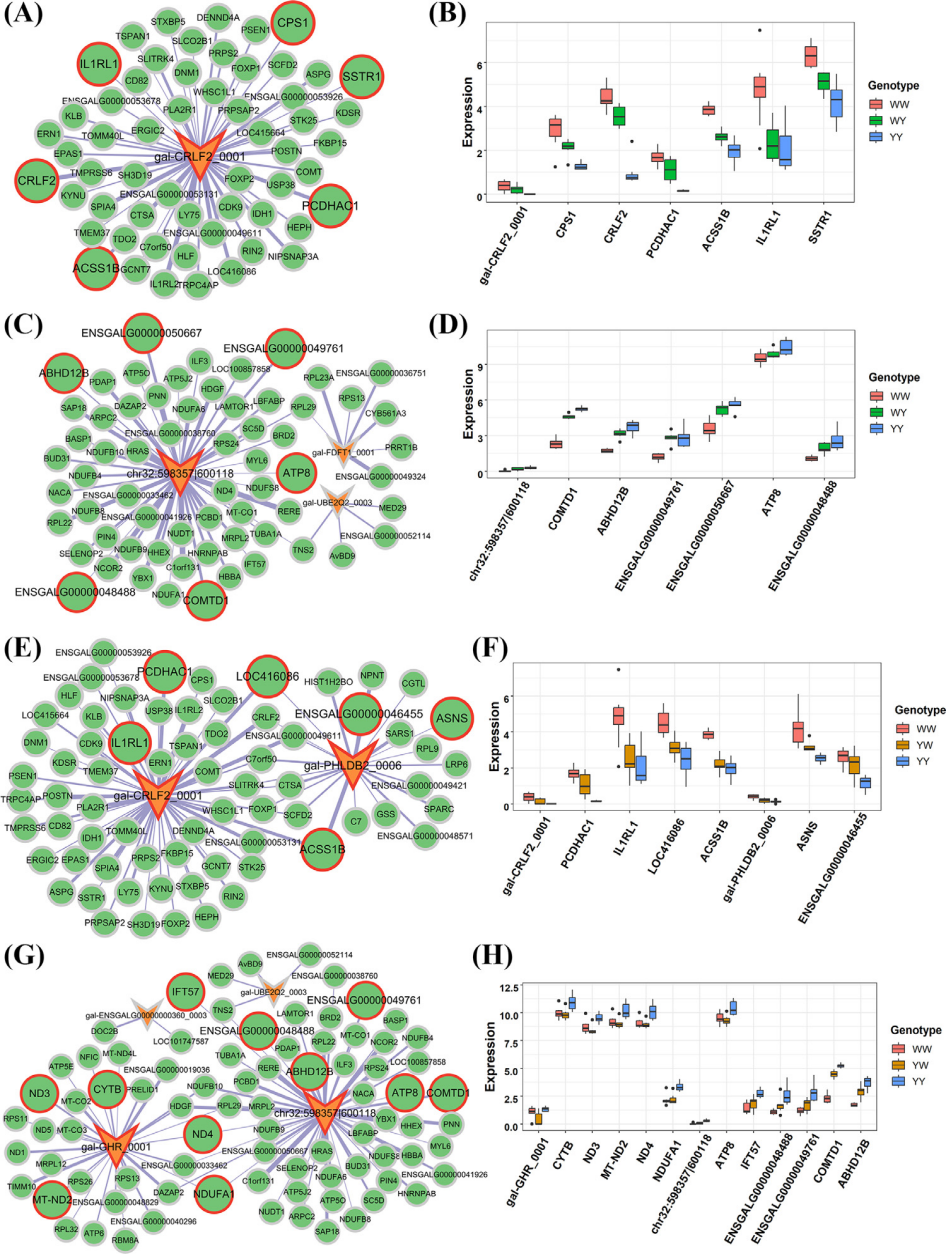
CircRNA-mRNA networks in the liver associated with heterosis for residual feed intake (**RFI**). CircRNA-mRNA networks in the blue (A) and yellow (C) module that associated with RFI for WY. The expression pattern of nonadditive circRNAS and genes in WY's blue (B) and yellow (D) module. CircRNA-mRNA networks in the blue (E) and yellow (G) module that associated with RFI for YW. The expression pattern of nonadditive circRNAS and genes in YW's blue (F) and yellow (H) module. In the networks, the circRNA and gene were plotted in “V” and “ellipse” shape, respectively. The shape with red border line denotes that the circRNA/gene is nonadditive expressed.

In the liver, nonadditive circRNAs were found in the MEblue and MEyellow for WY and that in the MEblue, MEturquoise and MEyellow for YW. In WY's MEblue, the nonadditive gal-CRLF2_0001 was correlated with 57 genes (Figure 6A), in which *CRLF2, CPS1, PCDHAC1* and *SSTR1* were dominantly expressed in a pattern same as gal-CRLF2_0001 (Figure 6B and Table S9b). For the yellow module in WY, 3 circRNAs were targeting 64 genes (Spearman's *r* > 0.6 and *P* < 0.05), and a novel circRNA ranging from 598,357 bp to 600,118 bp located in the cytochrome P450 2G1-like on the chromosome 32 (named gal-CYP2G1L_0001 here) was dominantly expressed (Figure 6C). The gal-CYP2G1L_0001 regulated the nonadditive expression of *COMTD1, ABHD12B, ATP8* and 3 unannotated genes (Figure 6D and Table S9b). In YW, gal-CRLF2_0001 and gal-CYP2G1L_0001 were also dominantly expressed in MEblue and MEyellow, respectively (Figures 6E and 6G). The expression level of gal-CRLF2_0001 was similar between WY and YW, closing to WW (Figures 6B and 6F), while the expression of gal-CYP2G1L_0001 showed contrary expression pattern between 2 crossbreds (pattern III for WY and pattern V for YW) (Figure 6H and Table S9b), which was consistent with the divergent RFI between the 2 crossbreds. Therefore, the gal-CYP2G1L_0001 was a promising regulator affecting the heterosis for RFI. In addition, we detected gal-PHLDB2_0006 and gal-GHR_0001 were dominantly expressed in the MEblue and MEyellow for YW, respectively (Figures 6E and 6G). The expression of gal-GHR_0001 of YW was significantly lower than that of YY, who was more efficient than YW and WW, suggesting the gal-GHR _0001 potentially modulated the heterosis for RFI. We further screened the nonadditive genes with same expression pattern as candidate circRNAs for 2 crossbreds. For the gal-CYP2G1L_0001, the targeted gene *COMTD1, ABHD12B, ENSGALG00000049761* and *ENSGALG00000050667* possessed same expression pattern (III) in WY. The nonadditive *NDUFA1, ND4, IFT57, ENSGALG00000048488* and *ENSGALG00000049761* expressed in same pattern (V) in YW (Table S9b). The *COMTD1, ABHD12B, ATP8* and *ENSGALG00000048488* were shared by 2 crossbreds. However, their expression patterns were same between 2 crossbreds, which was inconsistent with the divergent RFI between the crossbreds. *ENSGALG00000049761* was another common gene showing divergent expression similar to the expression pattern of gal-CYP2G1L_0001. For the gal-GHR_0001, the targeted gene *CYTB, ND3, ND2, ND4* and *NDUFA1* possessed same expression pattern (V) in YW (Table S9b). Interestingly, the *ND4* and *NDUFA1* involved in the oxidative phosphorylation pathway were interacted with both gal-CYP2G1L_0001 and gal-GHR_0001, which might be associated with heterosis for feed intake and efficiency in YW. Additionally, we detected 5 nonadditive circRNAs and 6 nonadditive genes uniquely expressed in YW, with same expression pattern (XI) in the turquoise module of liver tissue (Table S9b). Small RNA genes (*MIR6613, MIR6572*) suggested circRNAs are important regulators of non-coding RNAs expression. Although no modules were identified for feed intake and efficiency in the duodenum mucosa, pink and brown module were associated with traits at a level of *p* < 0.05 (Figure S2a). Furthermore, we found that YW's MEbrown harbored 2 nonadditive circRNAs chr5:52919904|52922537 (named gal_BRF1_0001 here) and chr8:14222295|14229099 (named gal_BCAR3_ 0001 here). The 2 circRNAs showed same expression pattern (IX) and were correlated with 213 genes, among which *HAAO, HEXB* and *ENSGALG00000045809* were nonadditive genes targeted by gal_ BCAR3_0001. The expression pattern of *HEXB* (IX) and *ENSGALG00000045809* (IX) were same as the expression pattern of gal_BCAR3_0001, while HAAO was expressed in pattern XI that consistent with RFI heterosis (Figure S3).

### The Function Mechanism for circRNAs Associated With Heterosis for Feed Efficiency

**Figure 7:**
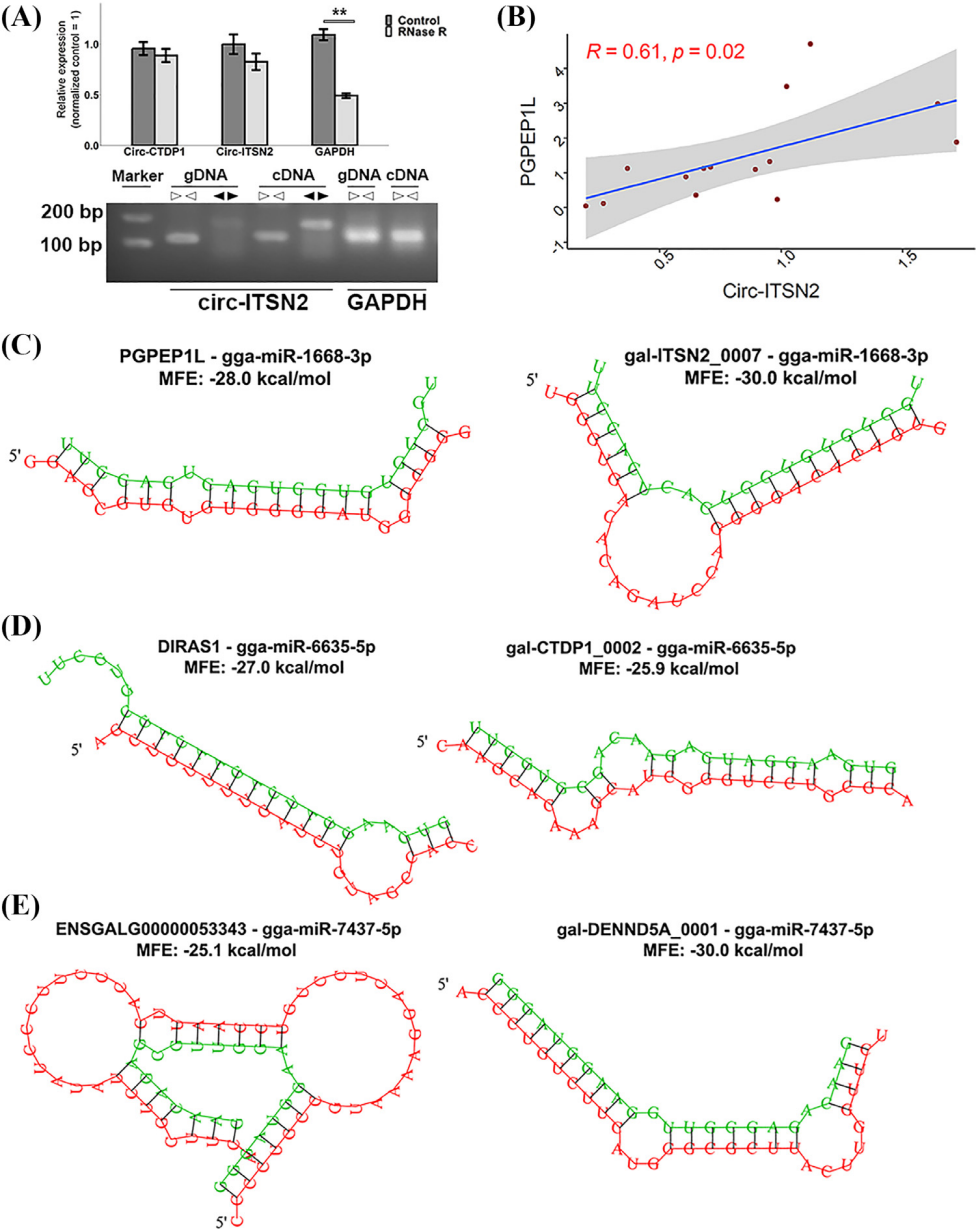
The validation and potential mechanism for circRNAs associated with heterosis for residual feed intake. (A) Validation of the stability and circularity of candidate circRNAs. Linear GAPDH mRNA was used as a negative control. (B) The correlation between circ-ITSN2 and PGPEP1L validated by qRT-PCR. (C) The predicted interaction among miR-1668-3p, circ-ITSN2 and PGPEP1L by RNAhybird software. (D) The predicted interaction among miR-6635-5p, circ-CTDP1 and DIRAS1 by RNAhybird software. (E) The predicted interaction among miR-7437-5p, circ-DENND5A and ENSGALG00000053343 by RNAhybird software.

From the results of WGCNA and expression pattern analysis, we clearly observed that circRNA-mRNA networks played a pivotal role in the formation of heterosis for feed intake and efficiency in YW, whose DFC and RFI was highly correlated, showing consistently negative heterosis (the crossbred YW consumed more feed and was more inefficient). We then validated the stability and circularity of gal-CTDP1_0002 and gal-ITSN2_0007 (Table 1) in the liver tissue, both of which were RNase R-resistant (Figure 7A). And the expression correlation between gal-ITSN2_0007 and *PGPEP1L* was highly positive (R = 0.61, *p* < 0.05), which could be considered candidate circRNA-gene pairs in the liver responded to the heterosis for feed intake and efficiency (Figure 7B). Given that circRNAs acted as miRNA sponges to facilitate gene expression, we proceeded to identify potentially feed efficiency-associated circRNA-miRNA-mRNA regulatory axes based on the candidate circRNA-mRNA pairs in the hypothalamus and liver (listed in Table 1). For the hypothalamus, we predicted 14 and 85 miRNAs (target score > 60) for 3′ UTR of *DIRAS1* and gal-CTDP1_0002 sequence, respectively. Gga-miR-6635-5p was a candidate miRNA that contacted the circRNA and gene, for which the minor free energy (MFE) of binding were -27.0 and -25.9 kcal/mol, respectively (Figure 7D). In the liver, we identified miRNAs that could be bound by the pair of gal-ITSN2_0007-*PGPEP1L* and gal-DENND5A_0001-*ENSGALG00000053343*. The most reliable miRNA was gga-miR-1668-3p with a binding MFE -28.0 and -30.0 kcal/mol for the gal-ITSN2_0007 and *PGPEP1L*, respectively (Figure 7C). Gga-miR-7437-5p was the only miRNA with target score > 60 for both gal-DENND5A_0001 and *ENSGALG00000053343* (Figure 7E). However, miRNAs were not found for genes involved in the oxidation phosphorylation pathway due to the short 3′ UTR sequence. Furthermore, internal ribosome entry site (**IRES**), which have been suggested to be potential mechanism for circRNA translation were predicted. Among these circRNAs, 3 circRNAs including gal-CYP2G1L_0001, gal-GHR_0001 and gal-GBE1_0002 (without predicted miRNAs) scored 0.58, 0.8 and 0.89, respectively (Table 2), suggesting that IRES-mediated cap-independent translation initiation was involved in these circRNAs’ function.

**Table 1:** The information of predicted miRNAs for candidate circRNA-mRNAs associated with heterosis for residual feed intake.

Tissue	Module	CircRNA	CircRNA position	Target gene	Group	Predicted microRNA	CircRNA score	Gene score
Hypothalamus	Brown	**gal-CTDP1_0002**	chr2:56435192|56446005	DIRAS1	YW	gga-miR-6635-5p	96	65
Liver	Yellow	gal-CYP2G1L_0001	chr32:598357|600118	ENSGALG00000049761	YW/WY	NO	–	–
				ND4	YW	NO	–	–
				NDUFA1	YW	NO	–	–
				IFT57	YW	NO	–	–
		gal-GHR_0001	chrZ:13478392|13495992	CYTB	YW	NO	–	–
				ND2	YW	NO	–	–
				ND3	YW	NO	–	–
				ND4	YW	NO	–	–
				NDUFA1		NO	–	–
	Turquoise	**gal-ITSN2_0007**	chr3:104232959|104235895	PGPEP1L	YW	gga-miR-6554-3p	93	98
						gga-miR-1668-3p	93	98
				ENSGALG00000051522	YW	gga-miR-12234-3p	54	79
		gal-GBE1_0002	chr1:96558455|96564165	ENSGALG00000051522	YW	NO	–	–
				LOC112532241	YW	NO	–	–
		gal-GBE1_0003		MIR6133	YW	NO	–	–
		gal-GCH1_0002	chr5:56539971|56542323	MIR6133	YW	NO	–	–
				MIR6572	YW	NO	–	–
		gal-DENND5A_0001	chr5:9696389|9705327	ENSGALG00000053343	YW	gga-miR-153-3p	53	68
						gga-miR-7437-5p	79	68

**Table 2:** The information of putative IRES for candidate circRNAs associated with heterosis for residual feed intake.

Tissue	Module	CircRNA	CircRNA position	Target gene	Group	CircRNA ORF length (aa)	IRES score
Hypothalamus	Brown	gal-CTDP1_0002	chr2:56435192∼56446005	DIRAS1	YW	184	0.82
Liver	Yellow	gal-CYP2G1L_0001	chr32:598357∼600118	ENSGALG00000049761	YW/WY	446	0.58
				ND4	YW	446	
				NDUFA1	YW	446	
				IFT57	YW	446	
		gal-GHR_0001	chrZ:13478392|13495992	CYTB	YW	316	0.8
				ND2	YW	316	
				ND3	YW	316	
				ND4	YW	316	
				NDUFA1		316	
	Turquoise	gal-ITSN2_0007	chr3:104232959|104235895	PGPEP1L	YW	–	0.73
				ENSGALG00000051522	YW	–	
		gal-GBE1_0002	chr1:96558455|96564165	ENSGALG00000051522	YW	156	0.89
				LOC112532241	YW	156	
		gal-GBE1_0003		MIR6133	YW	156	
		gal-GCH1_0002	chr5:56539971|56542323	MIR6133	YW	118	0.8
				MIR6572	YW	118	
		gal-DENND5A_0001	chr5:9696389|9705327	ENSGALG00000053343	YW	243	0.9
						243	

## DISCUSSION

The utilization of heterosis is the basis for crossbreeding in agriculture. Nowadays, the excellently commercial lines dominant the industry around the world. These lines are almost selected and cultivated through artificial crossbreeding. In the plant heterosis, substantial efforts have been made on the formation mechanism from the perspectives of classical genetics, parental genetic distance, quantitative trait loci, transcriptomes, proteomes, epigenetics (DNA methylation, histone modification and small RNA), and hormone regulation (Wu et al., 2021). Amuzu-Aweh et al. (2015) showed that it is possible to predict heterosis for egg production traits at the sire level by using genome-wide SNP data. The nonadditive genes involved in the oxidative phosphorylation were identified as the major genetic and molecular factors in the heterosis for chicken growth traits (Mai et al., 2021). Nevertheless, the understanding of the molecular basis for heterosis was far behind the utilization of it in chicken. In this study, we constructed a reciprocal cross population and sequenced the transcriptome for multiple feed efficiency-related tissues to analyze the differences between reciprocal crosses and their parents with divergent feed efficiency from the perspective of circRNA biology, aiming to explore the mechanism of heterosis formation in chicken.

As expected, we identified more circRNAs than that previously reported in the single tissue or cell type of chicken, such as skeletal muscle (Shen et al., 2019b), liver (Zhang et al., 2019), spleen (Wang et al., 2020), cecum (Yu et al., 2021), ovarian granulosa cells (Wang et al., 2022) and abdominal fat (Tian et al., 2021). But the number was smaller than that reported in granulosa cells (11,642 circRNAs) (Shen et al., 2019a) and embryonic muscle (13,377 circRNAs) (Liang et al., 2017). This might be due to the different algorithm, quality control criterions and number of samples among these studies. We also found that the circRNAs were mainly derived from the exonic regions, with fewer being derived from introns in the hypothalamus, liver, duodenum mucosa and ovary, which was consistent with the results of Shen et al., 2019a), who found that circRNAs of were mainly mapped to exons in the small yellow follicles, the smallest hierarchal follicles, and the largest hierarchal follicles ovarian follicles. In addition, the exon number, sequence length, and expression level of the identified circRNAs were consistent with previous researches in chicken using similar detection software and quality control standards (Shen et al., 2019a; Tian et al., 2021).

The number of identified circRNAs was significantly differed among 4 tissues, accompanying with the results of principal component analysis. We revealed that circRNAs were expressed in a tissue-specific manner, which were also detected in the multi-tissue of pig (Liang et al., 2017), mouse and human (Xia et al., 2017), and multiregion of human brain (Gokool et al., 2020). This was previously explained by the fact that the formation of circRNAs was regulated by tissue-preferential expressed RNA binding proteins (Ji et al., 2019), indicating that the uniquely regulatory roles of circRNAs in diverse organs were also existed in chicken. The low expression and the higher positive correlation compared to the negative correlation between circRNA and host gene were consistent with findings in the diverse tissues and cells (Shang et al., 2019), suggesting that the expression patterns of these circRNAs are not stochastic but instead are selectively maintained. The circRNAs mainly come from the circularization of exons and introns of host genes, which highlighted the specific role for each tissue. For example, the biological processes of neuro development and positive regulation of axonogenesis were only enriched in the hypothalamus. Progesterone-mediated oocyte maturation and GnRH signaling pathway were specifically enriched in the ovary. MAPK signaling pathway, mTOR signaling pathway and cellular senescence pathway that enriched by common host genes across 4 tissues were ubiquitous and reported to be modulated by circRNAs (Li et al., 2018; Gao et al., 2019; Haque et al., 2020). Single parent expression patterns are an extreme instance of gene expression complementation, which has been demonstrated to be highly correlated with mid-parent heterosis revealed by transcriptome analysis in plant (Baldauf et al., 2022). Our findings confirmed that the SPE circRNAs were highly tissue-specific and genotype-specific in both plant and animal (Li et al., 2021).

Nonadditive gene action was regarded as a specific expression pattern in crossbreds and could be the major force driving the formation of heterosis (Li et al., 2015). In the plant, researchers found that circRNAs were expressed at low levels with high specificity and identified 5 heterosis-related circRNAs using indica, japonica and their inter-subspecific hybrid rice as experimental materials (Wang and Wang, 2022). Zhang et al. (2023) also reported 7 circRNAs that positively or negatively regulate poplar growth heterosis. Combined with the WGCNA, we found the number of heterosis-related circRNAs was less than that of mRNAs and lncRNAs, indicating that circRNAs were essential to the formation of heterosis, but their effect was minor compared to mRNA and lncRNA (Shu et al., 2021). The candidate circRNA from the *CTDP1* (gal-CTDP1_0002) was previously documented to function as a competitive endogenous RNA (ceRNA) for miRNAs to regulate the expression of related genes and subsequently affected cancer cells (Li et al., 2020). Thus, we inferred that gal-CTDP1_0002 regulated the expression of *DIRAS1* by sponging the gga-miR-6635-5p in the hypothalamus. *DIRAS1* is widely expressed in the central nervous system and has been suggested to regulate acetylcholine release (Jeong et al., 2017), neural circuits of which play a role in the regulation of feeding behavior and intake (Wielaender et al., 2017). Gal-GHR_0001 was another candidate circRNA interacting with genes involved in the oxidative phosphorylation pathway in the liver. The potential mechanism was that gal-GHR_0001 encoded small peptide via IRES-mediated cap-independent translation initiation, supported by the IRES score of 0.8 and long ORF (Shang et al., 2019). How it enhances the expression of OXPHOS genes in the liver to regulate the formation of heterosis requires further analysis and experimental validation. Gal-ITSN2_0007 was demonstrated to target the miR-218-5p/LMO7 axis, promoting chicken embryonic myoblast proliferation and differentiation (Shen et al., 2021). Based on the interaction between gal-ITSN2_0007 and *PGPEP1L*, we also predicted multiple miRNAs could bind to both sequences of circRNA and gene. One of most reliable miRNAs was gga-miR-6554-3p that was previously reported to be associated with lipolysis by suppressing IRS1 gene in chicken (Ghafouri et al., 2021). Herein, gga-miR-6554-3p was predicted to targeted with *PGPEP1L*, which was demonstrated to enable pyroglutamyl-peptidase activity and involved in proteolysis. The pyroglutamyl-peptidase of tanycyte contributes to the hypothalamic control of basal metabolism (Sánchez et al., 2009), controlling resting metabolism, activity levels, and responses to external temperature and food intake. We suggested that the gal-ITSN2_0007 regulated expression of *PGPEP1L* by sponging the gga-miR-6554-3p in the liver. Further functional characterization of circRNAs in diverse tissues, cells and biological processes are needed to fully elucidate the role of circRNAs in chicken.

## CONCLUSIONS

In sum, we reported the first multi-tissue (hypothalamus, liver, duodenum mucosa and ovary) analysis of circRNA profiles based on comparison of 4 groups (2 reciprocal crosses and 2 parental lines) of 8 chickens each. We found tissue-specific and genotype-specific features of circRNAs in the crossbreds compared to the landscape of mRNA-lncRNA expression profile. The nonadditive expression pattern was dominant across tissues for these circRNAs in the crossbreds with divergent heterosis for feed intake and efficiency. The miRNA sponges and post-transcriptional role of circRNAs played on gene expression may actively contribute to heterosis for feed intake and efficiency, highlighting the novel role of circRNAs that played on the molecular basis of heterosis. The presence of circ-ITSN2 in the liver tissue was validated and predicted to affect the heterosis for residual feed intake by potentially targeting the *PGPEP1L* via gga-miR-6554-3p, which need further molecular validation in vitro and association analysis in multiple chicken population. Our findings add a new dimension to the exploration of core mechanisms underlying heterosis for feed intake and efficiency and could be used as fundamental information in genomic selection and mating in the investigated population.

## Appendix Supplementary materials

**Figure S2**. Weighted gene co-expression network analysis for the parental line and reciprocal crosses. The hierarchical cluster dendrogram and module-trait relationships were plotted for the duodenum mucosa (A) and ovary (B), respectively. Heatmap colors indicate positive/negative Pearson correlation coefficients. Correlation coefficients and *P*-values are shown within the cells (purple font, *P* < 0.01). MBW metabolic body weight, BWG body weight gain, DEM daily egg mass, DFC daily feed intake and RFI residual feed intake. Image, image 1

**Figure S3**. CircRNA-mRNA networks in the duodenum mucosa associated (0.01 ≤ *P* < 0.05) with heterosis for residual feed intake. (A) CircRNA-mRNA networks in the brown module that associated with RFI for YW. (B) The expression pattern of nonadditive circRNA and genes in WY. In the networks, the circRNA and gene were plotted in ‘V’ and ‘ellipse’ shape, respectively. The shape with red border line denotes that the circRNA/gene is nonadditive expressed. Image, image 2

**Table S1.** The expression patterns of differentially expressed circRNAs for hypothalamus, liver, duodenum mucosa and ovary. Image, image 3

**Table S2.** qRT-PCR was performed by primers (a), and PCR was performed by divergent and convergent primers (b). Image, application 4

**Table S3.** The summary of phenotypic value and their heterosis. Image, application 5

**Table S4.** The information for the predicted circRNAs. Image, application 6

**Table S5.** CircRNAs expreesed in at least half of all samples in the hypothalamus, liver, duodenum mucaosa and ovary. Image, application 7

**Table S6.** The circRNA and gene pairs with significant (P < 0.05) expression correlations in the hypothalamus, liver, duodenum mucosa and ovary. Image, application 8

**Table S7.** The expression pattern for the nonadditive circRNAs across 4 tissues in the crossbreds. Image, application 9

**Table S8.** Significant GO biological processes enriched by genes in the turquoise module for the liver. Image, application 10

**Table S9.** The nonadditive circRNAs and genes in the feed efficiency-related modules for hypothalamus (a) and liver (b), respectively. Image, application 11

Image, application 12
