# Supplementary figures and images for: Multi-tissue transcriptome profiling linked the association between tissue-specific circRNAs and the heterosis for feed intake and efficiency in chicken
Source: Poult Sci. 2024 Apr 20;103(7):103783. doi: 10.1016/j.psj.2024.103783 (PMC11091503; doi:10.1016/j.psj.2024.103783)

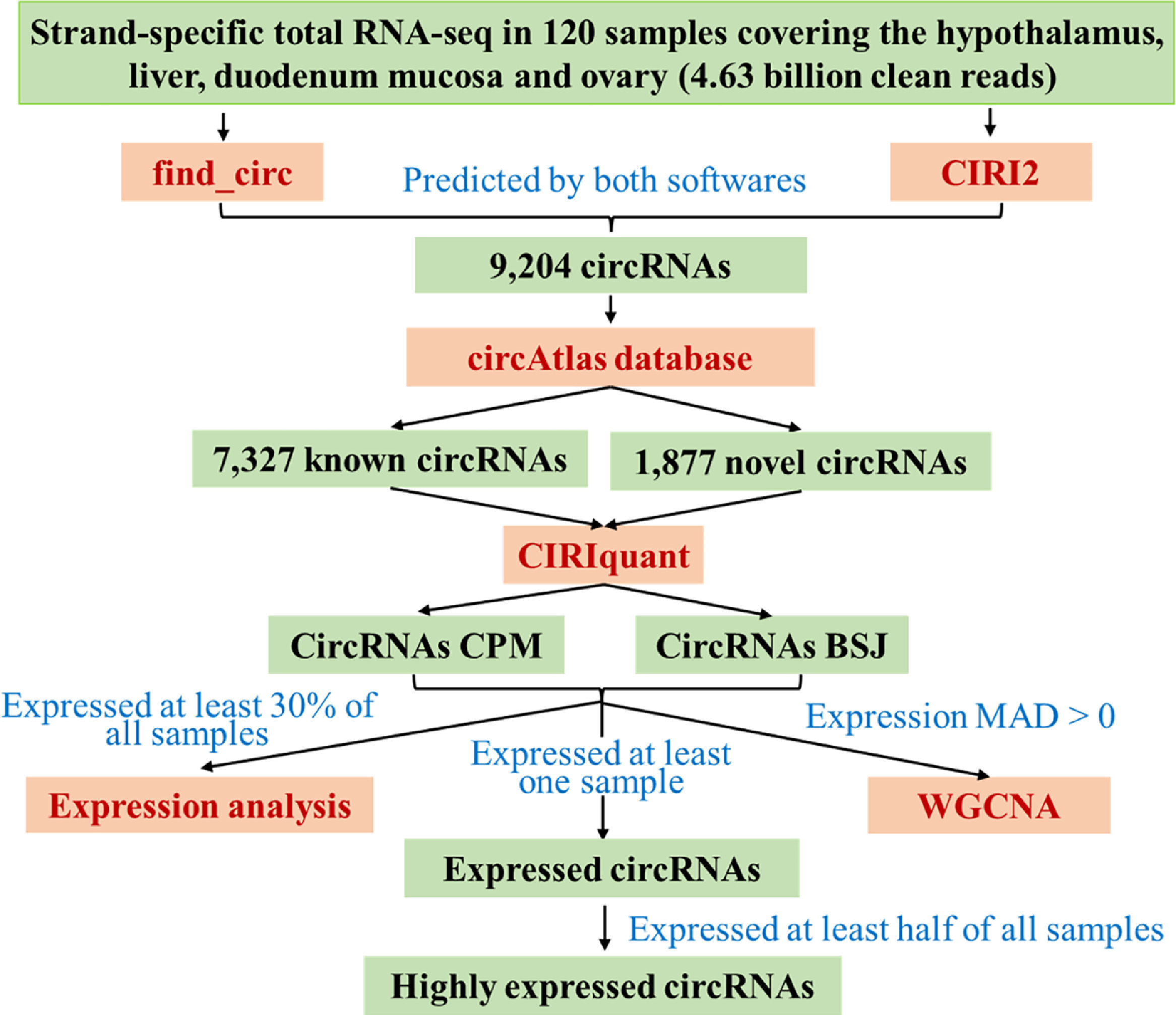

Supplement: Supplementary file 1 — Figure S2. Weighted gene co-expression network analysis for the parental line and reciprocal crosses. The hierarchical cluster dendrogram and module-trait relationships were plotted for the duodenum mucosa (A) and ovary (B), respectively. Heatmap colors indicate positive/negative Pearson correlation coefficients. Correlation coefficients and P-values are shown within the cells (purple font, P < 0.01). MBW metabolic body weight, BWG body weight gain, DEM daily egg mass, DFC daily feed intake and RFI residual feed intake. [file mmc1.jpg]

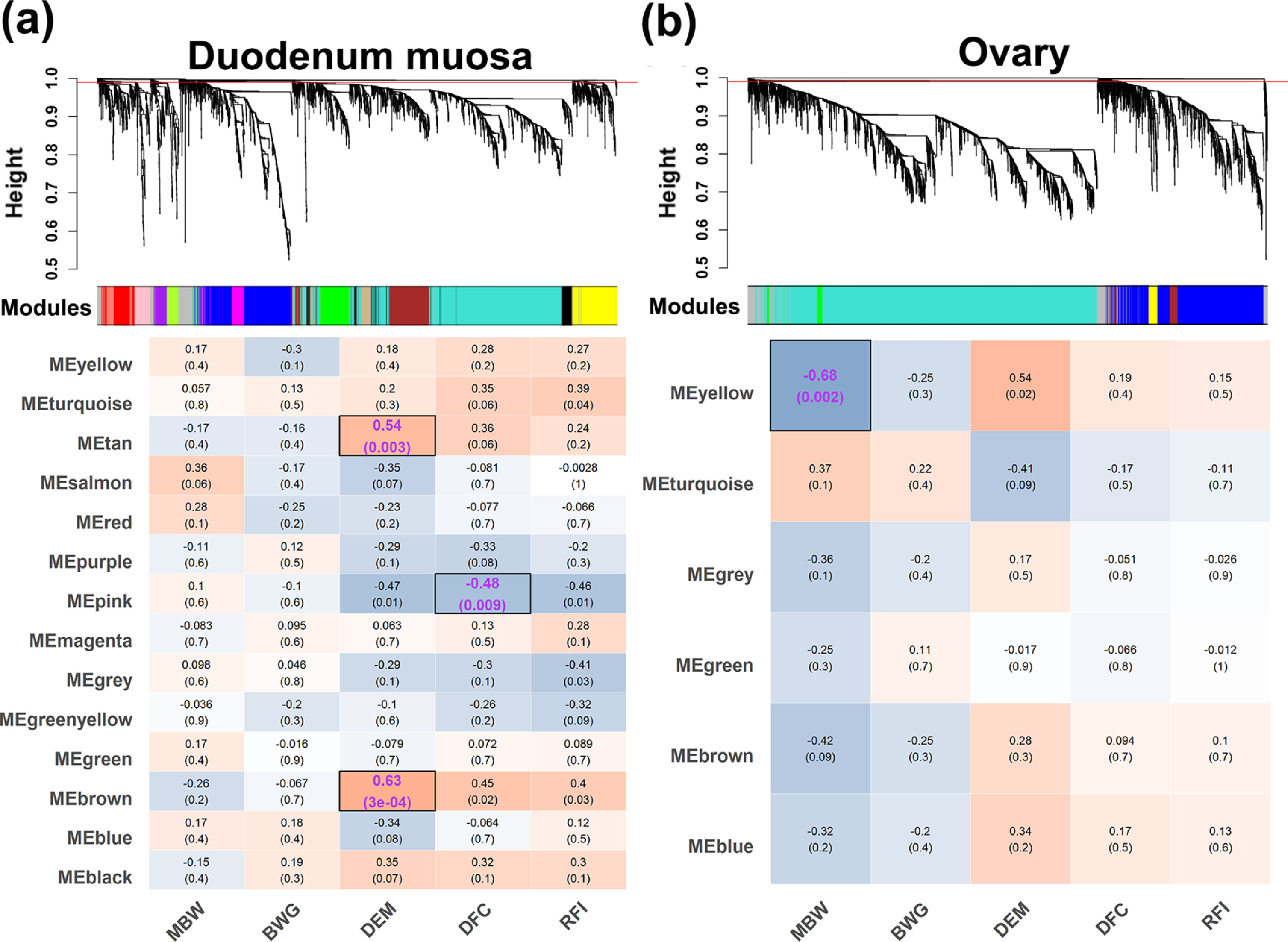

Supplement: Supplementary file 2 — Figure S3. CircRNA-mRNA networks in the duodenum mucosa associated (0.01 ≤ P < 0.05) with heterosis for residual feed intake. (A) CircRNA-mRNA networks in the brown module that associated with RFI for YW. (B) The expression pattern of nonadditive circRNA and genes in WY. In the networks, the circRNA and gene were plotted in ‘V’ and ‘ellipse’ shape, respectively. The shape with red border line denotes that the circRNA/gene is nonadditive expressed. [file mmc2.jpg]

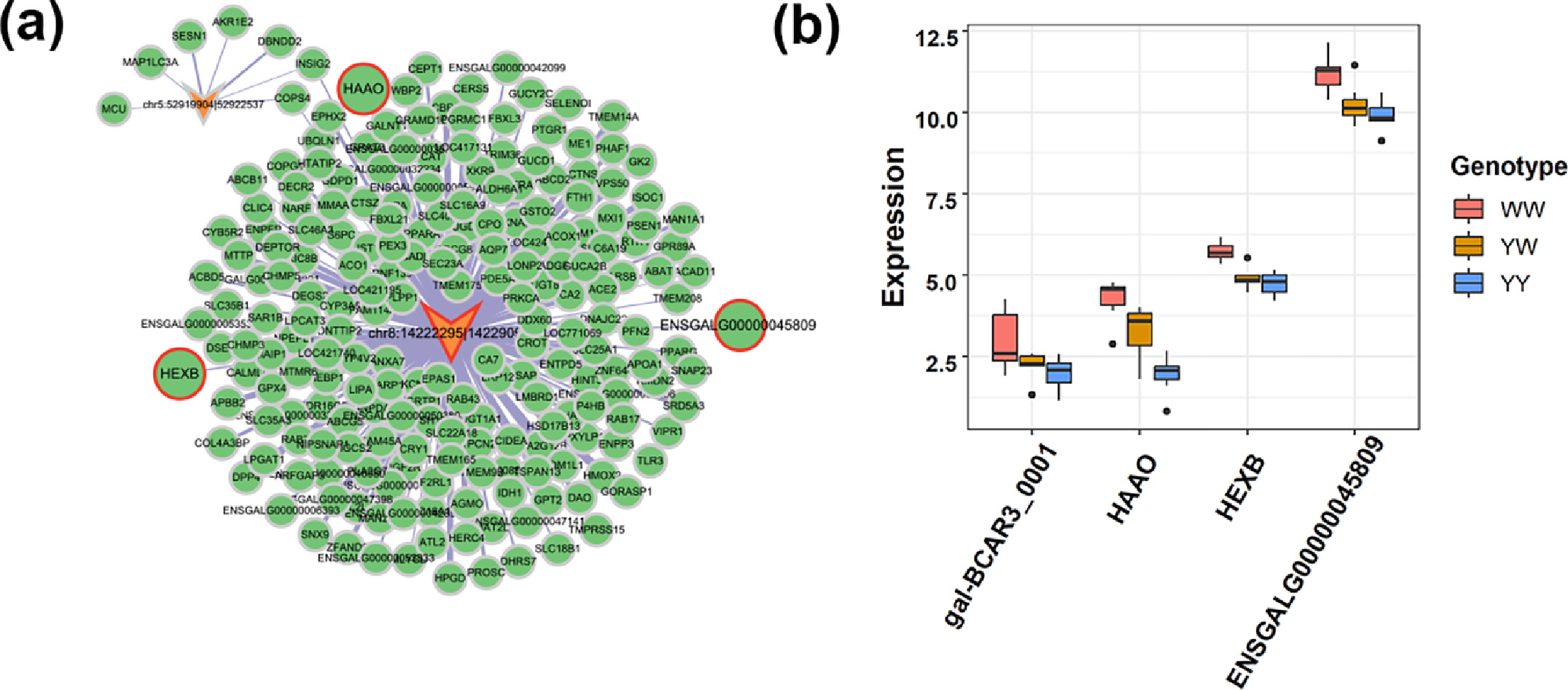

Supplement: Supplementary file 3 — Table S1. The expression patterns of differentially expressed circRNAs for hypothalamus, liver, duodenum mucosa and ovary. [file mmc3.jpg]
